# Synaptic Loss in Alzheimer's Disease: Mechanistic Insights Provided by Two-Photon *in vivo* Imaging of Transgenic Mouse Models

**DOI:** 10.3389/fncel.2020.592607

**Published:** 2020-12-17

**Authors:** Jaichandar Subramanian, Julie C. Savage, Marie-Ève Tremblay

**Affiliations:** ^1^Department of Pharmacology & Toxicology, University of Kansas, Lawrence, KS, United States; ^2^Axe Neurosciences, Centre de Recherche du CHU de Québec, Université Laval, Québec City, QC, Canada; ^3^Neurology and Neurosurgery Department, McGill University, Montreal, QC, Canada; ^4^Department of Molecular Medicine, Université Laval, Québec City, QC, Canada; ^5^Division of Medical Sciences, University of Victoria, Victoria, BC, Canada; ^6^Department of Biochemistry and Molecular Biology, The University of British Columbia, Vancouver, BC, Canada

**Keywords:** dendritic spines, microglia, two-photon, *in vivo* imaging, amyloid mouse models

## Abstract

Synapse loss is the strongest correlate for cognitive decline in Alzheimer's disease. The mechanisms underlying synapse loss have been extensively investigated using mouse models expressing genes with human familial Alzheimer's disease mutations. In this review, we summarize how multiphoton *in vivo* imaging has improved our understanding of synapse loss mechanisms associated with excessive amyloid in the living animal brain. We also discuss evidence obtained from these imaging studies for the role of cell-intrinsic calcium dyshomeostasis and cell-extrinsic activities of microglia, which are the immune cells of the brain, in mediating synapse loss.

## Introduction

Immunohistochemistry of *postmortem* brain from Alzheimer's disease (AD) patients revealed that synapse loss is the strongest correlate for the cognitive deficit (Terry et al., 1991; DeKosky et al., 1996; Scheff and Price, 2006; Scheff et al., 2007; de Wilde et al., 2016). Mouse models that express familial AD-associated mutations in genes coding for amyloid precursor protein and presenilin that increase amyloid levels in the brain or that express mutated Tau leading to neurofibrillary tangles provide an entry point to study mechanisms of synapse loss associated with prominent AD related pathologies, such as amyloid plaques and neurofibrillary tangles (Jankowsky and Zheng, 2017). Morphological and electrophysiological studies done on *ex vivo* and *postmortem* preparations from these mice have confirmed that human familial AD associated mutations cause synaptic dysfunction, ranging from impaired plasticity to increased loss of synapses (Selkoe, 2002; Knobloch and Mansuy, 2008; Spires-Jones and Knafo, 2012; Yu and Lu, 2012; Pozueta et al., 2013; Forner et al., 2017). *Postmortem* preparations only offer a snapshot of pathology that does not capture dynamic events that precede or follow the observed deficits. *Ex vivo* preparations, on the other hand, allow for monitoring dynamic events but not in the context of the intact neural circuitry of a living brain. In addition, the functions of microglia, which are the immune cells of the brain, are severely affected by experimental procedures (Hellwig et al., 2013; Gosselin et al., 2017).

The advent of fluorescence labeling technologies and two-photon microscopy enabled direct visualization of synapse dynamics *in vivo* in the living mouse brain (Grutzendler et al., 2002; Trachtenberg et al., 2002). Typically, neurons are sparsely labeled with a fluorescent cell fill to visualize the morphology of subcellular structures, such as dendritic spines (postsynapse) or axonal boutons (presynapse). Fluorescence labeling is achieved by expressing fluorescent proteins through transgene integration, viral delivery, or *in utero* electroporation. Optical access to fluorescently labeled neurons is achieved by replacing part of the skull with a glass coverslip or thinning it. Though two-photon excitation provides higher depth resolution than traditional single-photon excitation, it is still limited to ~450 μm from the surface of the brain (Takasaki et al., 2020). Therefore, non-invasive two-photon imaging studies are restricted to superficial layers of the cortex, however synaptic imaging in the hippocampus has been achieved using more invasive approaches, such as removal of overlying cortical tissue or micro-endoscopy (Mizrahi et al., 2004; Gu et al., 2014; Attardo et al., 2015). Two-photon imaging in mouse models of AD has allowed for the interrogation of synaptic dysfunction associated with amyloid and tau pathology in the intact circuitry of living animals (Tables 1, 2). More importantly, *in vivo* imaging of synapses allows for chronic monitoring of synaptic changes in the same neurons over time, thereby enabling the visualization of synaptic dynamics. In this review, we focus on how *in vivo* imaging using two-photon microscopy has revealed the properties and mechanisms of synapse loss in mouse models of AD, emphasizing mouse models of amyloidosis. Due to the depth limitations of two-photon imaging, most of these studies tracked synaptic changes in the somatosensory cortex (unless otherwise mentioned).

**Table 1:** Studies examining dendritic spine and neurite pathology in mouse models of amyloidosis using *in vivo* two-photon microscopy that are discussed in this review.

**Model strain**	**Method of neuronal labeling**	**Age at imaging**	**Brain region imaged**	**Interval between imaging sessions**	**Dendritic spine/bouton changes (compared to control)**	**Presence of neurite dystrophy**	**References**
PDAPP	Alexafluor-594-Dextran	19–22 months	Non-specified cortical regions	−	−	Yes	D'Amore et al., 2003
PSAPP	Crossed with Thy1-YFP transgenic mice	6 months	Non-specified cortex	2–4 days, 1–2 weeks, 4–5 weeks	Increased spine gain and loss but loss greater than gain	Yes	Tsai et al., 2004
Tg2576	Viral delivery of GFP	21–24 months	Somatosensory cortex	1 week	−	Yes	Spires et al., 2005
Tg2576	Viral delivery of GFP	8–10 months; 18–24 months	Somatosensory cortex	Minutes to 1 hour	Increased spine loss in 18–24 but not 8–10 months		Spires-Jones et al., 2007
Tg2576; PS1ΔE9; APP/PS1; PS1M146V	Viral delivery of a calcium indicator YC3.6	Tg2576: 17–20 months; PS1ΔE9: 5- 6 months; APP/PS1: 3–3.5 months.	Somatosensory cortex	−	Elevated intracellular calcium and disrupted calcium compartmentalization.	Yes	Kuchibhotla et al., 2008
APPswe/PS1d9; Tg2576; PDAPP	Crossed with Thy1-YFP transgenic mice	5−6 months	Non-specified cortex	One day or one week	−	Yes	Meyer-Luehmann et al., 2008
3xTg-AD	Crossed with Thy1-YFP transgenic mice	4-6, 8–10, 13–15 and 18–20 months	Somatosensory cortex	Few days	8–10 month - no change, 13–15 and 18–20 month - increased spine gain and loss	Yes	Bittner et al., 2010
APP1PS1	Viral delivery of GFP	6 month	Somatosensory cortex	−	−	Yes	Wu et al., 2010
APP PS1	Crossed with Thy1-YFP transgenic mice	3–4 months and 18–19 months	Somatosensory cortex	One week	Increased spine loss but same spine gain	−	Bittner et al., 2012
5X-FAD	Crossed with Thy1-YFP transgenic mice	3–4 months	Somatosensory cortex	Few days	Not reported.	No gross deficit observed.	Buskila et al., 2013
APP PS1 mice	Crossed with Thy1-GFP transgenic mice	3–4 months	Somatosensory cortex	One week	Increased spine gain and loss	−	Liebscher et al., 2014
APP23 and APPswe/PS1deltaE9	Crossed with Thy1-GFP transgenic mice	4–5 months	Somatosensory cortex	One week	Decreased spine gain and no change in loss.	−	Zou et al., 2015
APPPS1	Crossed with GAD1-GFP mice	4–11 months	Hippocampus	Weekly, monthly	Increased spine loss with age. Decreased spine stabilization after learning.	−	Schmid et al., 2016
APPswe/PS1deltaE9	Crossed with Thy1-GFP transgenic mice	4–5 months	Somatosensory cortex	One week	Enriched environment does not change spine turnover whereas it is increased in controls.	−	Zou et al., 2016
APP swe/PS1deltaE9	Viral delivery of calcium sensor GCaMP6	3 months	Primary motor cortex	Hours	Longer duration calcium transients in dendrites, decreased spine size in dendrites with long duration calcium currents.	−	Bai et al., 2017
APPswe/PSen1ΔE9	Crossed with Thy1-GFP transgenic mice	3–4 months; 10–11 months	Somatosensory cortex	2 weeks	Increase in both spine gain and loss.	−	Heiss et al., 2017
APPswe/PS1deltaE9 and APP-PS1	Crossed with Thy1-GFP transgenic mice	APPswe/PS1deltaE9: 7–13 months; APP-PS1: 3–10 months	Somatosensory cortex	One week	−	Yes	Blazquez-Llorca et al., 2017
APP-PS1	Crossed with VGLUT1^venus^ knockin mice	3–7 months	Somatosensory cortex	One week	Bouton density reduced closer to plaque	Yes	Peters et al., 2018
APP-PS1	Crossed with VGLUT1^venus^ knockin mice	3–6 months	Somatosensory cortex	One week	−	Yes	Peters et al., 2019
J20	Viral delivery of GFP	7–10 and 11–14 months	Somatosensory cortex	One week	Bouton but not spine density reduced	No	Stephen et al., 2019

**Table 2:** Studies examining dendritic spine and neurite pathology in mouse models of Tauopathy using *in vivo* two-photon microscopy that are discussed in this review.

**Model strain**	**Method of neuronal labeling**	**Age at imaging**	**Brain region imaged**	**Interval between imaging sessions**	**Dendritic spine/bouton changes (compared to control)**	**Presence of neurite dystrophy**	**References**
rTg4510	Crossed with Thy1-YFP transgenic mice	9–10 months	Somatosensory cortex	–	Reduced spine density	–	Kopeikina et al., 2013a
P301S Tau	Crossed with Thy1-YFP transgenic mice	4 months	Somatosensory cortex	3–4 days	Reduced spine density. Decreased spine gain and slightly decreased loss	–	Hoffmann et al., 2013
rTg4510	Viral delivery of calcium indicator YC3.6	8–9 months	Somatosensory cortex	–	Reduced spine density did not correlate with calcium levels in parent dendrite	–	Kopeikina et al., 2013b
rTg4510	Viral delivery of GFP	4, 5, and 6.5 months	Somatosensory cortex	One week	Reduced spine density. Both gain and loss are increased. Bouton turnover decreased.	–	Jackson et al., 2017
rTg4510	Viral delivery of GFP	4,5,6 and 7 months	Somatosensory cortex	One week	Reduced spine and bouton density. Spine turnover increased and bouton turnover decreased.	–	Jackson et al., 2017

## Amyloid Associated Neurite Pathology

After its development for *in vivo* imaging (Grutzendler et al., 2002; Trachtenberg et al., 2002), two-photon microscopy was quickly adopted to visualize neurite dystrophy associated with amyloidosis (D'Amore et al., 2003). The initial study confirmed findings from *postmortem* AD patient brains (Onorato et al., 1989) and amyloid mouse models (Richardson and Burns, 2002) that plaques alter the trajectory of neurites and are associated with their dystrophy (D'Amore et al., 2003). Consistently, many subsequent *in vivo* imaging studies confirmed the existence of dystrophic neurites (variably defined as swelling >2.5 μm or a volume 2-fold over normal neurites) near plaques (Tsai et al., 2004; Brendza et al., 2005; Spires et al., 2005; Spires-Jones et al., 2007, 2011; Kuchibhotla et al., 2008; Meyer-Luehmann et al., 2008; Bittner et al., 2010, 2012; Wu et al., 2010; Zou et al., 2015, 2016; Schmid et al., 2016; Blazquez-Llorca et al., 2017; Peters et al., 2018). The presence of dystrophic neurites near plaques could either mean that plaques cause neurite dystrophy or dystrophic neurites promote plaque formation. Addressing this question requires monitoring the dynamics of neurites and plaques over time, which is not possible with traditional imaging approaches on *postmortem* brain slices. Longitudinal imaging of dystrophic neurites and plaques shows that plaque formation precedes dystrophic neurites (Meyer-Luehmann et al., 2008; Blazquez-Llorca et al., 2017; Peters et al., 2018). In one of the mouse models, APPswe/PS1E9 mice, dystrophic dendrites were present in regions with no apparent plaques (Meyer-Luehmann et al., 2008). Longitudinal imaging of these regions did not identify any *de novo* plaque formation near the dystrophic dendrites. Interestingly, during this time, some of the neurites returned to normalcy or disappeared. Only 60% of the dystrophic neurites remained stable over 2 weeks (Meyer-Luehmann et al., 2008). If dystrophic neurites do not promote plaque formation, it is more likely that plaques increase the abundance of dystrophic neurites. This possibility is supported by the observations that the appearance of dystrophic neurites follows plaque appearance (Meyer-Luehmann et al., 2008; Blazquez-Llorca et al., 2017). The curvature of neurites across the plaque continues to increase following plaque formation, with damaged neurites appearing in the subsequent days (Tsai et al., 2004; Meyer-Luehmann et al., 2008). Only ~25% of neurites near plaque developed dystrophy, and more detailed analyses of their dynamics revealed that they are highly stable with a mean lifetime of 76 days (Blazquez-Llorca et al., 2017). The volume of dystrophy is highly variable, and interestingly, is also highly dynamic, with dystrophies undergoing both shrinkage and expansion (Blazquez-Llorca et al., 2017). Plaques smaller than 4 μm are not associated with dystrophic neurites, and as the plaque size increased, the extent of dystrophy also increased (Blazquez-Llorca et al., 2017; Peters et al., 2018).

Though dystrophic neurites do not appear to promote plaque formation, they may still contribute to the growth of preformed plaque. Plaque growth occurs over a long period, exhibiting sigmoidal growth kinetics (Christie et al., 2001; Yan et al., 2009; Burgold et al., 2011, 2014; Condello et al., 2011; Hefendehl et al., 2011; Bittner et al., 2012). One of the contributing factors for plaque growth is the local concentration of the enzyme BACE-1, whose cleavage of amyloid precursor protein results in amyloid peptides present in the plaque (Hussain et al., 1999; Sinha et al., 1999; Vassar et al., 1999; Yan et al., 1999; Lin et al., 2000). BACE-1 levels are increased in dystrophic neurites in cultured neurons and brain sections of AD patients, and 5XFAD amyloid mouse model (Zhang et al., 2009; Kandalepas et al., 2013; Sadleir et al., 2016), and therefore, dystrophic neurites can contribute to plaque growth. Consistent with this idea, inhibition of BACE-1 decreased the growth rate of plaques (Peters et al., 2018), which, in turn, reduced the formation of dystrophic neurites associated with plaque (Peters et al., 2018). The vicious cycle of amyloid plaque growth, the formation of neurite dystrophy, and accumulation of BACE-1 in dystrophic neurites also results in the formation of satellite plaques (Peters et al., 2018). Interestingly, the deletion of the gene coding for microtubule binding protein Tau reduces the accumulation of BACE-1 in dystrophic neurites and reduces the formation of satellite plaques (Peters et al., 2019). Thus, *in vivo* two-photon imaging approaches have allowed us to understand the kinetics of plaque formation and growth and its relevance to the appearance of dystrophic neurites in a manner not feasible with traditional *postmortem* analyses.

## Amyloid Associated Dendritic Spine Pathology

Dystrophic neurites near plaques have a reduced density of dendritic spines (Spires et al., 2005). Since neurite dystrophy is increased after plaque appearance, is the reduction in synapses triggered only after plaque formation? One of the consensuses from the different *in vivo* imaging studies performed in mouse models of amyloidosis is that the density of dendritic spines is lower within 50 μm from the plaque compared to farther away from the plaque or non-transgenic controls (Spires-Jones et al., 2007; Kuchibhotla et al., 2008; Zou et al., 2015) but see Stephen et al. (2019). Though less pronounced, a reduction in spine density (Spires et al., 2005; Bittner et al., 2010) or a reduction in mature spine morphology (Zou et al., 2015) compared to non-transgenic controls was observed in dendrites > 50 μm away from plaque. A smaller reduction in spine density farther away from plaque could be due to reduced local concentration of soluble amyloid compared to the vicinity of a plaque. The reduction in spine density did not correlate with the size of the plaque (Spires et al., 2005), supporting the idea that soluble amyloid in the periphery of the plaque rather than the plaque itself is responsible for synapse loss (Mucke et al., 2000; Koffie et al., 2009). Consistently, spine loss is observed in *ex vivo* preparations following exposure to amyloid peptides (Hsieh et al., 2006; Shrestha et al., 2006; Shankar et al., 2007), and *in vivo* 1 day after the injection of soluble amyloid in non-transgenic mice (Arbel-Ornath et al., 2017).

Soluble amyloid is present even in the absence of plaques; therefore, if soluble amyloid alone were sufficient for spine loss, one would expect a significant reduction in spine loss prior to plaque formation. Longitudinal imaging of spines before and following *de novo* plaque formation reveals that spine density begins to reduce only 4.5 weeks after plaque formation (Bittner et al., 2012). Consistently, multiple imaging studies show normal spine density before plaque formation (Spires-Jones et al., 2007; Kuchibhotla et al., 2008; Bittner et al., 2010). Though these observations tend to support a role for amyloid plaque itself in spine loss, a more parsimonious explanation is that spine loss requires a high enough concentration of amyloid peptides that becomes available at an age when plaques are formed in the strains used for *in vivo* imaging (Maia et al., 2013). The less dramatic effect of soluble amyloid in transgenic mouse models of amyloidosis on overt spine loss *in vivo* compared to *ex vivo* preparations indicates possible resistance to spine loss when the buildup of amyloid is gradual compared to a sudden spike used in bath applications of *ex vivo* preparations. In addition, the lack or alteration of clearance mechanisms, such as microglial phagocytosis (Mandrekar et al., 2009), may exacerbate the synaptotoxic effect of amyloid in *ex vivo* preparations. These explanations, however, are at odds with the findings that brain slices from some mouse models of amyloidosis show synapse loss prior to plaque formation (Hsia et al., 1999; Moechars et al., 1999; Mucke et al., 2000; Lanz et al., 2003; Jacobsen et al., 2006). One plausible explanation for this discrepancy is that the brain regions examined for synapse loss using *in vivo* imaging (mostly, somatosensory cortex) and brain slices (mostly, hippocampus) are not the same. The vulnerability of synapses or the local concentration of amyloid could differ between brain regions and contribute to the plaque dependence for spine loss in brain regions imaged using two-photon microscopy. However, this may not be the sole reason because, in 3xTg-AD mouse strain, spine loss was observed in brain slices of the hippocampus and frontal cortex only after plaque formation, though these areas did not exhibit a characteristic plaque-distance dependence for spine loss (Bittner et al., 2010).

Pre- and postsynaptic terminals may exhibit differential plaque distance dependent vulnerability. The evidence for the vulnerability of presynaptic terminals in amyloid mouse models has been contradictory. Some histological studies found evidence for the loss of synaptophysin (Rutten et al., 2005; Dong et al., 2007; Tampellini et al., 2010) whereas others have not (King and Arendash, 2002; Rutten et al., 2003; Boncristiano et al., 2005; Hong et al., 2016), even in the same amyloid mouse model. *In vivo* imaging of presynaptic boutons in amyloid model mice show increased dynamics of boutons near the plaque compared to farther away from the plaque (Liebscher et al., 2014; Blazquez-Llorca et al., 2017; Stephen et al., 2019). The plaque distance-dependent synaptic loss and dystrophy holds up even when a presynaptic protein (Vglut1) is directly visualized *in vivo* (Peters et al., 2018). One of the reasons for the lack of consensus with respect to loss of presynaptic terminals in amyloid models could be due to variations in the sampling of different cell types between studies.

The plaque distance dependence of synapse loss observed in *in vivo* imaging studies using amyloid mouse models is in stark contrast to the observed loss of synapses affecting both pre- and postsynaptic elements across *postmortem* cortex (Terry et al., 1991; DeKosky et al., 1996; Sze et al., 1997; Scheff and Price, 2006; Scheff et al., 2007; de Wilde et al., 2016), despite plaques occupying only 5–10% of cortical volume in AD patients (Terry, 2000). Multiple differences between AD patients and mouse models of amyloidosis can account for the difference in plaque distance-dependent effect on synapses. One remarkable feature of mouse models of amyloidosis is that they exhibit limited or no neuronal death (Wirths and Bayer, 2010; Jankowsky and Zheng, 2017). The death of a neuron whose axons travel far will be accompanied by synapse loss farther from the soma. Thus, the lack of synapse loss farther away from plaque could be a consequence of limited or no plaque-associated cell death in these mouse models. Another possibility for preferential loss of synapses closer to plaques is that most mouse models of amyloidosis do not display the Tau pathology observed in human AD patients. When Tau mutation is combined with amyloidosis, as in the triple transgenic 3xTg-AD mice, spine loss was observed closer to and farther away from the plaque (Bittner et al., 2010).

## Spine Loss and Cognitive Decline

If spine loss increases only at the age when plaques are present, then one would expect cognitive deficits to be apparent only after plaque formation. However, cognitive deficits are observed before plaque formation in many amyloid mouse models (Holcomb et al., 1998; Wisniewski and Sigurdsson, 2010). In one of the strains, APP23, where cognitive decline precedes plaque formation, it was found that amyloid precursor protein accumulates intracellularly, and the amount of intracellular accumulation correlates with spine loss (Zou et al., 2015). Another caveat in reconciling cognitive studies with *in vivo* spine imaging studies is that most *in vivo* imaging studies in amyloid mouse models are restricted to the somatosensory cortex. Typically used cognitive tests, such as spatial or contextual memory tasks, may not elicit synaptic remodeling in the somatosensory cortex. In contrast, changes to sensory experience have been shown to cause synaptic remodeling in the somatosensory cortex (Trachtenberg et al., 2002). In the one of the amyloid mouse models, synaptic remodeling elicited by exposure to an enriched environment is disrupted in the somatosensory cortex before plaque appearance when spine density is normal (Zou et al., 2016; Heiss et al., 2017).

One of the main reasons most *in vivo* imaging studies are restricted to the somatosensory cortex is that it is easily accessible for non-invasive imaging. Imaging hippocampus, located ~1 mm deep in the brain, is out of the range for conventional two-photon imaging. Two-photon imaging of dendritic spines in the hippocampus is achieved by microendoscopy or by removing the overlying cortex (Mizrahi et al., 2004; Gu et al., 2014; Attardo et al., 2015). To date, to our knowledge, spine imaging *in vivo* in the hippocampus has not been performed in excitatory neurons of amyloid mouse models. However, a group of inhibitory neurons in the hippocampus, positive for somatostatin, that possess dendritic spines, has been studied using *in vivo* two-photon imaging in an amyloid mouse model (Schmid et al., 2016). The density of spines on these interneurons is reduced near the plaque. This reduction is due to a decrease in input from cholinergic neurons, which are lost due to cell death. In these somatostatin positive interneurons, new spines are formed in response to fear conditioning; however, this process is disrupted in the amyloid model (Schmid et al., 2016).

Most *in vivo* imaging studies use dendritic spines as a proxy for excitatory synapses. The maturation status of the spine is inferred from their shape, stability, and age. Newly formed spines are usually thin and are transient. If they persist for more than 4 days, they are highly likely to carry synapses (Knott et al., 2006). Mature persistent spines resemble mushroom-like structures and are called mushroom spines (Bourne and Harris, 2007). Though age, shape, and stability of dendritic spines could be a good predictor for the maturation status of a synapse, they are not entirely reliable. Spines that persist for 4 days may not contain synaptic proteins associated with mature synapses (Subramanian et al., 2019). Even if the plaque-associated spine loss observed in the superficial cortical regions is globally true, structural alterations, such as changes to AMPA receptors concentration, not captured by imaging spine alone could underlie cognitive decline. Therefore, synaptic abnormalities associated with cognitive decline may not require overt spine loss and the mechanisms could differ depending on the brain region and the model strains.

## Mechanisms of Amyloid Associated Spine Loss

In adult mice, spine formation and elimination are balanced to maintain spine density (Villa et al., 2016; Subramanian et al., 2019). A reduction in the density of spines under amyloid pathology could result from the decreased formation of new spines or increased elimination of preexisting spines or both. No consensus has emerged on whether amyloid pathology triggers spine reduction by regulating formation or elimination. *In vivo* imaging studies have found increased spine elimination with no change in formation or smaller increase in formation (Tsai et al., 2004; Spires-Jones et al., 2007; Bittner et al., 2012), decreased spine formation with no change or an increase in elimination (Zou et al., 2015), or an increase in both formation and elimination (Bittner et al., 2010; Liebscher et al., 2014; Heiss et al., 2017). How could an increase in both formation and elimination result in reduced spine density? Newly formed dendritic spines could mature into stable synapses or disappear without forming synapses (Subramanian et al., 2019). If amyloid pathology reduces synapse stabilization, neurons will continue to make futile attempts to form synapses, whereas under non-pathological conditions, due to synapse stabilization, there would be fewer futile attempts. Each futile attempt will be counted as a dynamic event, and therefore, APP mice would have higher spine dynamics and yet have lower spine density.

How could amyloid pathology induce neurite dystrophy and spine loss? Calcium signaling regulates synaptic plasticity and dendritic arbor development (Konur and Ghosh, 2005), and growing evidence suggests that amyloid pathology disrupts neuronal calcium homeostasis (Holscher, 1998; Kawahara, 2004; Small, 2009; Brawek and Garaschuk, 2014; Popugaeva et al., 2017). Consistent with *ex vivo* preparations (Mattson et al., 1992; Guo et al., 1999; Demuro et al., 2005; Smith et al., 2005; Mattson, 2007), *in vivo* imaging of calcium reveals that dendrites closer to plaque have increased intracellular neuronal calcium in the intact brain of amyloid mouse models (Kuchibhotla et al., 2008). Before plaque formation, dendritic calcium levels in an amyloid mouse model are not different from non-transgenic mice (Kuchibhotla et al., 2008). In non-transgenic mice, calcium concentration within the spine does not correlate with that of the parent dendrite, whereas under amyloid pathology, there is a linear relationship between the two, suggesting that the compartmentalization of calcium in spines is lost (Kuchibhotla et al., 2008). Amyloid peptides can increase intracellular calcium within an hour after exposure, but spine loss occurs only 24 h later (Arbel-Ornath et al., 2017). Interestingly, spines that shrink following the application of amyloid peptide in the motor cortex are the ones that were activated during the prolonged dendritic calcium current elicited by the amyloid peptide (Bai et al., 2017).

## A Role for Tau in Synapse Loss

The *in vivo* imaging studies described thus far are mostly focused on how amyloidosis associated with familial AD cases influence synapses. The effect of accumulation of hyper-phosphorylated Tau, another prominent AD-related pathology, on synaptic structure integrity is relatively less studied using *in vivo* two-photon imaging. The effect of overexpressing mutant Tau on synapse density has yielded mixed results in the histological analysis (Shahani et al., 2006; Eckermann et al., 2007; Yoshiyama et al., 2007; Hoover et al., 2010; Rocher et al., 2010; Crimins et al., 2011; Jaworski et al., 2011; Kremer et al., 2011; Alldred et al., 2012). In contrast, there is consensus among *in vivo* two-photon imaging studies that dendritic spine density is reduced in Tau mouse models (Bittner et al., 2010; Hoffmann et al., 2013; Kopeikina et al., 2013a,b; Jackson et al., 2017, 2020). The first *in vivo* imaging study describing the effect of Tau on synapses was done in triple transgenic 3xTg-AD mice that also had mutations associated with amyloidosis (Bittner et al., 2010). However, later studies using Tau models also showed a decrease in spine density. In a Tau model with P301S mutation, spine gain is reduced and is not matched by an equivalent reduction in spine loss, whereas, in the control mice, formation and elimination of spines are balanced. As a consequence, spine density is reduced in the Tau mutant model. Interestingly, the spine deficits are present at an age when no neurofibrillary tangles are apparent, and Tau itself is not localized to dendritic spines (Hoffmann et al., 2013). *In vivo* imaging also revealed decreased spine density in 8–9 month-old rTg4510 mouse model with P301L mutation. Surprisingly, in the same study, array tomography revealed no difference in synaptic density compared to wild-type control mice (Kopeikina et al., 2013a). Later *in vivo* imaging studies using this model confirmed a progressive decline in spine density between 4 and 6.5 months of age (Jackson et al., 2017, 2020). The decline in spine density is not uniform in all dendrites, with some dendrites exhibiting complete loss of spines. Higher turnover of dendritic spines, in some instances, is followed by the loss of associated dendritic branches. Over 6 months, ~35% of the dendrites are lost. In contrast, the axonal loss is preceded by a decreased turnover of boutons. The loss of synapses in this model also occurred at an age when neurofibrillary tangles are not yet present (Jackson et al., 2017, 2020). Interestingly, unlike amyloid models, spine loss in the rTg4510 model is not associated with elevated intracellular neuronal calcium (Kopeikina et al., 2013b).

Multiple cellular pathologies, such as intracellular calcium homeostasis, mitochondrial dysfunction, energy metabolism, and reactive oxygen species, associated with amyloidosis, have been imaged *in vivo* using two-photon microscopy (McLellan et al., 2003; Xie et al., 2013; Arbel-Ornath et al., 2017; Bai et al., 2017; Gomez et al., 2018; Lerdkrai et al., 2018; Calvo-Rodriguez et al., 2020). However, the dynamics of synapse loss in relation to these different cellular deficits resulting from amyloidosis remains to be explored by *in vivo* two-photon imaging. Synapse loss may not occur solely due to these cell-intrinsic factors. Growing evidence indeed suggests that microglial phagocytosis may be a key player in synaptic loss in amyloid and/or Tau mouse models. Below, we discuss how two-photon *in vivo* imaging has also uncovered a novel role for microglia during amyloid and Tau pathology.

## Microglial Implication in AD: Insights from Two-Photon *in vivo* Imaging Studies

Microglia, which are the resident innate immune cells of the brain, have been intimately associated with AD since the disease was first described. In his landmark studies, Dr. Alois Alzheimer described glial cells developing excess fibers and containing “adipose saccules” (Alzheimer et al., 1995). Over 80 years later, studies conducted on *postmortem* AD brain samples revealed that microglia surrounding plaques express elevated levels of histocompatibility antigens and the pro-inflammatory cytokine IL-1 (Rogers et al., 1988; Griffin et al., 1989; Overmyer et al., 1999). Ultrastructural studies further uncovered microglia's intimate relationship with plaques, and even posited that microglia were responsible for amyloid deposition (Wisniewski et al., 1989; Perlmutter et al., 1990). Though it was later discovered that neurons produce amyloid precursor protein and amyloid (Wisniewski et al., 1989; Alzheimer et al., 1995), the role of microglia in AD has remained largely elusive. Over the past 30 years, microglia in the AD brain have vacillated between valiant protectors, powerless observers, and indiscriminate destroyers. In this section of the review, we cover the insights into their roles in synaptic loss that were provided using two-photon *in vivo* imaging (Table 3) and complementary techniques.

**Table 3:** Studies examining microglial interactions with neuronal or synaptic loss in mouse models of amyloid or Tau pathology using *in vivo* two-photon microscopy that are discussed in this review.

**Model strain**	**Method of neuronal labeling**	**Age at imaging**	**Brain region imaged**	**Interval between imaging sessions**	**Neuronal/synaptic changes (compared to control)**	**Presence of neurite dystrophy**	**Reference**
3xTg-AD	Crossed with CX3CR1-GFP and Thy1-YFP transgenic mice	4–6 months	Somatosensory cortex	7 days	Reduced neuronal loss in the absence of CX3CR1 (knockout)	−	Fuhrmann et al., 2010
5xFAD	Crossed with Thy1-YFP and CX3CR1-GFP	10–12 months	Somatosensory cortex	5 days	Increased neurite dystrophy in the absence of microglial coverage	Yes	Condello et al., 2015
APP-PS1	Crossed with Thy1-YFP and CX3CR1-iDTR to deplete microglia	>12 months	Motor, somatosensory, and visual cortices	7 days	Increased loss of spines and shaft atrophy associated with amyloid plaques during microglial depletion	Yes	Zhao et al., 2017

## Loss of Microglial Phagocytic Abilities in AD

Two-photon *in vivo* studies in the APP/PS1Δe9 and Tg2576 models demonstrated that removing microglia during the chronic disease stage causes significant growth of existing plaques (Zhao et al., 2017). Combining these data with the fact that microglia (including the dark microglia, a subset identified by its markers of cellular stress including the condensation state of its cytoplasm and nucleoplasm resulting in a dark appearance under electron microscopy; Bisht et al., 2016) are occasionally seen containing amyloid deposits led to conclude that while microglia may be competent phagocytes, they are unable to control amyloid levels late during AD pathology. Of note, the dark microglia are rare in the healthy mature brain, but increase in number up to 10-fold with pathological conditions that include chronic stress, aging and amyloid deposition (in APP/PS1Δe9 mice; Bisht et al., 2016). This microglial subset discovered with electron microscopy displays hyper-ramified processes that extensively ensheath and engulf synaptic elements (pre- and postsynaptic), suggesting their role in the pathological remodeling of neuronal circuits in AD (Stratoulias et al., 2019; St-Pierre et al., 2020).

While neuroinflammation is a main hallmark of AD, alongside amyloid deposition and tangle formation, prolonged exposure to pro-inflammatory cytokines inhibits microglial phagocytic ability (Koenigsknecht and Landreth, 2004). Two-photon imaging in APP/PS1Δe9 mouse slices demonstrated that microglia lose their mobility and phagocytic capabilities as plaque deposition increases (Krabbe et al., 2013). Slice culture studies further determined that microglia from aged APP/PS1Δe9 and 5XFAD mice have reduced phagocytic capacity, possibly due to an impaired MerTK signaling (Savage et al., 2015). Work by others has determined that acute exposure to amyloid does not affect microglial phagocytic competence, whereas prolonged exposure in later disease stages results in reduced phagocytosis (Wendt et al., 2017). Together these data suggest that while microglial response to amyloid may be beneficial in the short-term, chronic exposure to amyloid may stunt microglia and prevent them from performing their normal phagocytic duties required to clear the brain from toxic or inflammatory debris.

Environmental enrichment has been shown using two-photon *in vivo* imaging to increase microglial amyloid clearing capacities thus preventing prion-like seeding of amyloid in a mouse model, while also reversing the deficits in neurogenesis and memory (Ziegler-Waldkirch et al., 2018). Environmental enrichment is well-known to promote beneficial neuroprotective microglial activities (Savage and Tremblay, 2019). However, increasing microglial phagocytosis with other strategies should be undertaken with extreme caution, as microglia could phagocytose the incorrect cargo. The same complement-mediated pathway which prunes excess synapses during normal brain development causes mistargeted phagocytosis of synapses early in mouse models of amyloid pathology, before plaques are deposited (Hong et al., 2016). Inhibiting C1q, C3, or microglial complement receptor CR3 in amyloid mouse models was shown to prevent synaptic loss, as well as cognitive impairment (Hammond et al., 2019). The microglial receptor TREM2, which displays various genetic variants in AD and is expressed by dark microglia, was also shown to mediate synaptic pruning during normal development, in cooperation with astrocytes (Filipello et al., 2018; Jay et al., 2019). Determining the outcome of TREM2 gene loss, haplodeficiency or variants on microglia-mediated synaptic loss in mouse models of amyloid and Tau pathology is an important topic of investigation (Yuan et al., 2016; Gratuze et al., 2020).

Similarly, fractalkine signaling between the neuronal chemokine fractalkine and its unique receptor CX3CR1, which is expressed by microglia, is a main mode of neuron-microglia communication in the brain. During normal physiological conditions, fractalkine signaling plays key roles in synaptic maturation, pruning, and plasticity, and mediates the adaptation of the brain and behavior to environmental challenges (Paolicelli et al., 2014; Tay et al., 2017). In AD pathology, evidence from complementary techniques revealed that fractalkine signaling is detrimental during amyloid pathology, yet beneficial in Tau pathology (Lee et al., 2010; Chen et al., 2016; Bemiller et al., 2018). *In vivo*, two-photon imaging indicates that 3xTg-AD mice deficient in fractalkine signaling are protected from neuronal loss, contrary to 3xTg-AD controls with an intact fractalkine signaling (Fuhrmann et al., 2010). In the 3xTg-AD mice, microglia were also shown to display an increased process velocity around the disappearing neurons, suggesting a possible involvement in their elimination, at early stages of pathology still devoid of plaques and Tau pathology (Fuhrmann et al., 2010). Similarly, using two-photon *in vivo* imaging in a zebrafish model of Tau pathology, in which neurons express hTau^P301L^, microglia were recently shown to transform their morphology (retraction and reduced number of processes, enlarged cell bodies), while increasing their migration and phagocytic activity toward dying neurons. Nevertheless, microglial phagocytosis failed in this context to remove all the dying neurons (Hassan-Abdi et al., 2019).

## Emergence of Neuroprotective Phenotypes

While microglia are not sufficient to prevent AD pathology, synaptic loss, and cognitive impairment, they nonetheless form a barrier around plaques and slow disease progression, at least early on during disease pathogenesis. A major avenue of therapeutic research is focused on restoring proper microglial metabolism and physiological function, thus enabling them to clear amyloid, remove apoptotic neurons as well as provide trophic support for synaptic maintenance. Two-photon *in vivo* imaging of healthy mice revealed that microglia play an important role in the formation of dendritic spines, through their secretion of brain-derived neurotrophic factor, which is required for motor learning (Parkhurst et al., 2013). Microglia were also well-shown to dynamically contact pre- and postsynaptic elements by two-photon *in vivo* imaging during normal physiological conditions, and these interactions were frequently followed by the elimination of dendritic spines during experience-dependent plasticity (Tremblay et al., 2010). Whether microglia could exert similar beneficial roles at synapses in AD remains to be determined.

Recent studies identified a number of neuroprotective microglial phenotypes present on a subset of microglia within the AD brain, and these provide further potential for specific, microglia-targeted therapeutics. Two-photon *in vivo* imaging in 5XFAD mice revealed that microglia “wall off” plaques and likely promote neuronal and/or synaptic survival in regions affected by the amyloid pathology (Condello et al., 2015). In fact, dystrophic neurons were much more commonly seen in regions near plaques that were not covered by microglia, as these regions contained increased levels of toxic amyloid oligomeric and protofibrillar species. Microglial ability to seal off amyloid plaques from the surrounding neuropil is dependent on TREM2, as knockout mice had significantly reduced plaque area covered by microglia, while displaying increased plaque-associated neuronal dystrophy (Yuan et al., 2016).

Recent technical advances in the field now allow researchers to study populations of microglia from specific brain microenvironments, and thus characterize differences between microglia associated with plaques and those from nearby brain regions. These studies have begun to uncover the specifically and differentially-regulated transcriptome of microglial cells associated or not with plaques. When plaque-associated microglia were microdissected from amyloid mouse models, transcriptomic analysis identified increased genes associated with priming (i.e., immunological alert) and cellular metabolism as well as lysosomal activity (Kamphuis et al., 2016; Yin et al., 2017). While two-photon *in vivo* imaging of the primed microglia in particular and their dynamics at synapses is currently lacking, these studies indicate that plaque-associated microglia are not dystrophic or senescent, but rather highly active cells attempting to digest the amyloid, as supported by ultrastructural studies (El Hajj et al., 2019). These data support the idea that microglia surrounding plaques prevent plaque growth by removing oligomeric and protofibrillar amyloid from their immediate surroundings.

Additional work using single-cell RNAseq uncovered a disease-associated microglial (DAM) subtype in amyloid mouse models and human brain tissues (Keren-Shaul et al., 2017). A similar subtype was subsequently named the microglia-associated with neurodegeneration (MGnD) (Krasemann et al., 2017). These disease-associated subsets lose homeostatic microglial markers and upregulate genes associated with phagocytosis of apoptotic cells. The authors posit that MGnD microglia require contact with, if not phagocytosis of, dystrophic neurons like those associated with plaques, while depending on Trem2 and Apoe expression (Krasemann et al., 2017). These disease-associated phenotypes echo the ultrastructurally-defined dark microglia, but further *in vivo* research is required to determine the independent or concerted implication of these different microglial types in synaptic loss.

## Conclusion and Perspectives

*In vivo* imaging approach using two-photon *in vivo* microscopy has found that dendritic spines are destabilized by the extracellular deposition or intracellular accumulation of amyloid. Cell intrinsic factors, such as elevated calcium, and extrinsic factors, like microglial phenotypic transformation elicited by the amyloid or Tau pathology, disrupt dendritic spine stability and might specifically trigger synaptic loss. Future two-photon imaging studies to simultaneously visualize the dynamics of axon terminals, dendritic spines, synaptic proteins, intracellular calcium, and microglia, together with amyloid and Tau pathology, *in vivo* in mouse models, will provide novel insights into the dynamic events preceding and causing loss of pre- and postsynaptic elements.

## Author Contributions

JS and M-ÈT designed and wrote the manuscript. JCS contributed to the section on microglia. All authors contributed to the article and approved the submitted version.

## Conflict of Interest

The authors declare that the research was conducted in the absence of any commercial or financial relationships that could be construed as a potential conflict of interest.
